# Q&A with Editorial Board Member Professor Jun Lu

**DOI:** 10.1038/s42004-023-00815-7

**Published:** 2023-01-13

## Abstract

Professor Jun Lu talks about the driving force for his career pathway and his academic journey from chemical physics to materials science and electrochemistry, as well as his experience of being an Editorial Board Member for *Communications Chemistry*.

Jun Lu is chair professor at Zhejiang University, China. He received his bachelor degree in chemical physics from University of Science and Technology of China (USTC) in 2000. He completed his Ph.D. in materials science from the Department of Metallurgical Engineering at University of Utah in 2009. Following a DOE-EERE postdoctoral fellowship under the Vehicles Technology Program, he joined the Division of Chemical Sciences and Engineering at Argonne National Laboratory as chemist in 2015. As a scientist, Dr. Lu has been committed to solving some of the world’s most challenging problems on the rechargeable Li-ion battery (LIB) and the next generation Li batteries. In the past 10 years, he has built up world-leading research programs in batteries, where he pioneered and led the research on novel cationic and anionic redox (CAR) materials for high energy density LIBs, Li-O2 and Li-S batteries. His current research interests focus on electrochemical energy storage and conversion technology, with the main focus on beyond Li-ion battery technologies. He was awarded the first DOE-EERE postdoctoral fellowship as part of the Vehicles Technology Program from 2011-2013. He was elected Associate President and Board Committee Member of the International Academy of Electrochemical Energy Science (IAOEES). He was also the inaugural awardee of the IAOEES Award for Research Excellence in Electrochemical Energy in 2016. In 2022, he received ECS Battery Division Technology Award, Research Excellence Award in Electrochemical Energy Storage (EES Award), ACS Energy & Fuel (ENFL) Division, and IBA Research Award.

**Figure:**
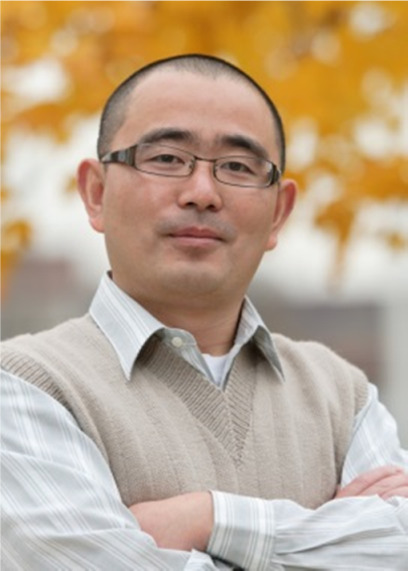
Jun Lu

Why did you choose to be a scientist?

Curiosity has been the biggest driving force for me to choose my career pathway, and being a scientist is how to get your curiosity most fulfilled. I enjoy eureka moments as much as being bewildered and taking the arduous journey to problem solve. But becoming a scientist didn’t cross my mind until I spent time at University of Science and Technology of China to get my bachelor degree. There I met many likeminded people who are exemplary scientists.

What scientific development are you currently most excited about?

Nuclear fusion comes at the top of my list. As a layman in nuclear physics, the recently reported “ignition” stage of nuclear fusion is pretty exciting to me. Harnessing nuclear energy is still a long way to go but I think we can see light at the end of the tunnel where environmental pollution is behind us. Parallel to nuclear energy, artificial intelligence technology is in its golden age. I am thrilled by the auto driving cars whenever I see one, and also constantly curious about what new chemistry/material AI can bring us by the massive screening of the existing data.

What direction do you think your research field should go in?

Electrochemistry has a specific functionality—energy conversion and storage. As the global environment worsens and the recent abnormal climate causes damage to the world, I believe the need for renewable energy and corresponding energy storage technology is more pressing than ever.

What attracted you to becoming an Editorial Board Member for *Communications Chemistry*?

As a chemist, the only way to keep learning and generate good ideas is to connect myself to a greater community. I decided to become an Editorial Board Member for *Communications Chemistry* because it is a team of talented scientists publishing research that interests me the most, and also, I had confidence that the benefits would be reciprocal to both me and the community.

What have you gotten out of the experience of being an Editorial Board Member for *Communications Chemistry*?

Being an EBM has made me meet more scientists, way more than I expected. Communication with these talented people from different research areas is truly satisfying and intriguing, which made me re-evaluate my research from a different perspective and engender new research ideas.

How do your editorial responsibilities integrate with your academic role?

Good academic research is to answer a question that is well rooted in reality, and my editorial responsibilities trained me to spot such questions consciously. It forces me to look at the bigger picture, what other scientists are interested in, and in what area my electrochemical expertise can contribute.

What do you see as the role of *Communications Chemistry* in the scientific community?

*Communications Chemistry* broadens the possibility for every member of the scientific community. It embraces intriguing ideas and significant research, and guarantees authors a wide range of readers with its open access policies. Scientific researchers with a desire for state-of-the-art research ideas, or looking for cross-disciplinary research applications, shall find suitable objects to read from *Communications Chemistry*.

*This interview was conducted by the editors of Communications Chemistry*.

